# The efficacy of machine learning models in forecasting treatment failure in thoracolumbar burst fractures treated with short-segment posterior spinal fixation

**DOI:** 10.1186/s13018-024-04690-3

**Published:** 2024-04-01

**Authors:** Neda Khaledian, Seyed Reza Bagheri, Hasti Sharifi, Ehsan Alimohammadi

**Affiliations:** 1 Kermanshah University of Medical Sciences, Kermanshah, Iran; 2 Department of neurosurgery, Kermanshah University of Medical Sciences, Imam Reza hospital, Kermanshah, Iran; 3 Clinical Research Development Center, Taleghani and Imam Ali Hospitals, Kermanshah University of Medical Sciences, Kermanshah, Iran

**Keywords:** Thoracolumbar burst fractures, Short segment posterior spinal fixation, Failure of treatment, Machine learning models, Load sharing classification

## Abstract

**Background:**

Although short-segment posterior spinal fixation (SSPSF) has shown promising clinical outcomes in thoracolumbar burst fractures, the treatment may be prone to a relatively high failure rate. This study aimed to assess the effectiveness of machine learning models (MLMs) in predicting factors associated with treatment failure in thoracolumbar burst fractures treated with SSPSF.

**Methods:**

A retrospective review of 332 consecutive patients with traumatic thoracolumbar burst fractures who underwent SSPSF at our institution between May 2016 and May 2023 was conducted. Patients were categorized into two groups based on treatment outcome (failure or non-failure). Potential risk factors for treatment failure were compared between the groups. Four MLMs, including random forest (RF), logistic regression (LR), support vector machine (SVM), and k-nearest neighborhood (k-NN), were employed to predict treatment failure. Additionally, LR and RF models were used to assess factors associated with treatment failure.

**Results:**

Of the 332 included patients, 61.4% were male (*n* = 204), and treatment failure was observed in 44 patients (13.3%). Logistic regression analysis identified Load Sharing Classification (LSC) score, lack of index level instrumentation, and interpedicular distance (IPD) as factors associated with treatment failure (*P* < 0.05). All models demonstrated satisfactory performance. RF exhibited the highest accuracy in predicting treatment failure (accuracy = 0.948), followed by SVM (0.933), k-NN (0.927), and LR (0.917). Moreover, the RF model outperformed other models in terms of sensitivity and specificity (sensitivity = 0.863, specificity = 0.959). The area under the curve (AUC) for RF, LR, SVM, and k-NN was 0.911, 0.823, 0.844, and 0.877, respectively.

**Conclusions:**

This study demonstrated the utility of machine learning models in predicting treatment failure in thoracolumbar burst fractures treated with SSPSF. The findings support the potential of MLMs to predict treatment failure in this patient population, offering valuable prognostic information for early intervention and cost savings.

## Background

Thoracolumbar fractures are frequently occurring injuries to the spine [1, 2], with burst fractures—impacting both the anterior and mid columns of the spine, per the Denis three-column classification—constituting about 20% of these fractures [3, 4]. Managing these fractures has sparked significant debate [5–7], with various studies proposing different approaches, including conservative treatment, anterior surgery, posterior surgery, or a combination of both [7, 8].

Several studies have shown positive outcomes for short-segment posterior spinal fixation (involving instrumentation one level above and one level below the fractured area) in carefully selected cases [8, 9]. Restricting the number of instrumented levels offers advantages such as reducing the risk of adjacent segment disease and limitations in range of motion [5].

Despite the favorable outcomes linked to short-segment posterior spinal fixation in thoracolumbar fractures, there is a notable incidence of treatment failure, with reported implant failure rates ranging from 9 to 54% for this approach [1, 8]. Factors contributing to treatment failure may include the absence of anterior support and biomechanical failure due to inadequate instrumentation [2, 7].

Nonetheless, despite these considerations, short-segment posterior spinal fixation remains the preferred choice for the majority of thoracolumbar fractures due to its favorable clinical results.

Machine learning models (MLMs) have emerged as a novel approach for assessing the impact of various independent variables. Traditional MLMs conduct computations to uncover patterns and relationships within specific data sets, excelling at generating algorithms without relying on equations, thus enabling predictions based on adaptable relationships between data points [10]. Recently, MLMs have found application in biostatistics and medicine for categorizing and forecasting patient outcomes [11, 12]. Despite the potential of improved performance over traditional statistical modeling in handling large data sets, there is limited published research on predicting factors associated with treatment failure in thoracolumbar burst fractures treated with SSPSF. The current study aims to evaluate the effectiveness of MLMs in predicting these factors.

## Methods

We conducted a retrospective evaluation of 332 consecutive patients who underwent short segment posterior spinal fixation (SSPSF) for single-level traumatic thoracolumbar burst fractures at our center between May 2016 and May 2023. Patients with pathologic/osteoporotic fractures, a history of previous surgery, or multiple vertebral fractures were excluded from the study. Approval for this study was obtained from the Scientific Research Board of the Kermanshah University of Medical Sciences, and all patients provided informed written consent before enrollment.

Upon admission to the emergency department, all patients underwent a thorough physical examination, and the intensity of back pain was assessed using the visual analogue scale (VAS). Imaging studies, including anteroposterior and lateral thoracolumbar radiography, thoracolumbar CT scans, T1- and T2-weighted MRI images, and short-tau inversion-recovery (STIR) sequences, were performed for all patients to evaluate the integrity of the posterior ligamentous complex (PLC).

The severity of injury was calculated using the Thoracolumbar Injury Classification and Severity Score (TLICS), with cases scoring more than 4 being considered candidates for surgery. Additionally, the Load Sharing Classification (LSC) was calculated for each patient, considering factors such as vertebral body comminution, kyphosis correction after surgery, and collapse of the vertebral body in the sagittal plane.

We also assessed various radiological parameters, including the Cobb angle, percentage of anterior height compression (PAHC), interpedicular distance (IPD), vertebral body compression rate (VBCR), and canal compromise.

The Cobb angle was determined by measuring the angle formed between the two tangents of the upper and lower endplates of the vertebrae above and below the fracture [13].

Canal compromise was assessed by calculating the ratio of the spinal canal diameter at the index level to the average of the spinal canal diameter at the vertebrae above and below the fractured vertebra [14].

The interpedicular distance (IPD) was evaluated by comparing the distance between the pedicles of the index vertebrae with the distance between the pedicles of the adjacent vertebrae above and below the fracture.

VBCR (Vertebral Body Compression Ratio) was computed as the ratio of the anterior vertebral height of the fractured vertebra to the posterior vertebral height of the fractured vertebra, multiplied by 100%. PAHC (Percentage Anterior Height Compression) was calculated as the anterior vertebral height of the fractured vertebra divided by the average of the anterior vertebral height of the vertebra above and below the fracture, multiplied by 100% [15, 16].

The failure of treatment was defined as the presence of instrument failure and/or progressive kyphosis during the follow-up period. Patients were categorized into two groups (failure of treatment and non-failure of treatment), and potential risk factors for treatment failure were compared between these groups.

### Statistical analysis

We utilized SPSS 23 software (SPSS Inc., Chicago, Illinois) for data analysis. The data was presented as mean ± standard deviation. To compare continuous and categorical variables between the failure of treatment and non-failure of treatment groups, we employed the Student’s t-test and the Chi-square test, respectively. Additionally, a binary logistic regression analysis was conducted to evaluate factors associated with treatment failure. The significance level for all analytical tests was set at < 0.05.

## Model development

In this study, we employed four machine learning models, namely random forest (RF), logistic regression (LR), support vector machine (SVM), and k-nearest neighborhood (k-NN), to predict failure of treatment in thoracolumbar burst fractures treated with SSPSF. Additionally, LR and RF models were utilized to assess factors associated with the failure of treatment. Each model underwent training before evaluation. The dataset was divided into two sets – a training set and a test set – at an 80:20 ratio. The training set was utilized to fit the models, while the test set was used to evaluate the models’ performance. Feature selection was based on significance in univariate analysis, with features exhibiting significance in the univariate analysis considered as input for the machine learning methods.

## Decision tree (DT) and random forest (RF) models

A decision tree (DT) is a tree-like structure used to make decisions based on presented data, with the root node representing the question to be addressed. Each node is linked via branches to subsequent child nodes by determining the best-split feature obtained by the split criterion. The binary DT separates each parent node into two subsets (child nodes), with binary divisions continuing until all observations are classified, leading to a leaf (terminal node) or outcome. Random forest (RF) is a machine learning ensemble consisting of several DTs. Each tree independently predicts the outcome and votes for the corresponding class. RF assigns the outcome to the class with the most votes, relying on a consensus of multiple trees to make more accurate predictions through its ability to capture complex relationships. In this study, 500 DTs were used to create the RF model, known for its capacity to manage intricate data and alleviate overfitting in classification and regression. The process of developing RF is well illustrated in existing literature [17].

## Logistic regression (LR)

Logistic regression (LR) is a widely used predictive model for making clinical decisions and is commonly employed in the classification of binary outcomes. The LR algorithm creates a sigmoid curve to represent the relationship between inputs and an outcome, mapping inputs to probabilities (between 0 and 1) that describe the likelihood of belonging to one of two classes. By using the logistic regression model, calculating the probability of each data point belonging to either outcome is easily attainable. After determining the probability of each person belonging to each class, each person is assigned to the group with the highest probability.

## Support vector machine (SVM)

The support vector machine (SVM) is a machine-learning algorithm used for both regression and classification, and it finds applications in chemometrics, bioinformatics, and biometrics [12]. Its core principle involves creating an optimal decision boundary, represented as a line, to separate data points and minimize error. In a two-dimensional plane, each dimension corresponds to an attribute or feature, while each observation is depicted as a data point. The algorithm aims to create a hyperplane, or best line, that effectively separates one group of points from another in a linear fashion. When the data is linearly separable, hyperplanes with maximum margins between one group of points and the hyperplane are better suited for making accurate predictions [18]. In cases where the data is not linearly separable, a kernel function is used to map the data to a higher-dimensional space, allowing linear separation without altering the original data. In this study, the radial basis function (RBF) kernel function, known for its high generalizability, was employed [19, 20].

## K-Nearest neighbors (K-NN)

The k-nearest neighbors (k-NN) is a straightforward, supervised machine learning algorithm utilized in both classification and regression. Its objective is to assign a data point to a class based on the nearest point in the training dataset. Among the nearest neighbors, the class with the highest number of occurrences is considered predictive. In regression, the average value of its neighbors is used. The steps of the k-NN algorithm for classifying new data are as follows: determining the number of nearest neighbors (k), computing the distance between the new data and training data points, ranking the distances, and finally classifying the new data based on the majority of votes from these neighboring points [21, 22].

## Performance criteria

The performance of the predictive models was evaluated using performance criteria, including accuracy, sensitivity, specificity, positive predictive value (PPV), and negative predictive value (NPV). Additionally, the area under the curve (AUC) of the receiver operating characteristic (ROC) was employed to assess the models’ ability to predict the failure of the treatment.

## Software

For statistical analysis, SPSS version 23 software was utilized to present descriptive and inferential statistics, as well as to conduct univariate and multivariate analyses. The random Forest package [23] was used to fit the RF model, the e1071 package [24] for fitting SVM, and the caret package [25] for calculating performance criteria. These packages are accessible in R4.0.3 software.

## Results

Out of a total of 332 subjects who underwent SSPSF for treating thoracolumbar burst fractures, 204 (61.4%) were males and 128 (38.6%) were females. The average age and follow-up time were 47.49 ± 9.75 years and 20.57 ± 5.31 months, respectively. The most common causes of trauma were traffic road accidents (54.5%) and falls from height (31.9%). T12 (38.3%) and L1 (33.7%) were the most frequently affected vertebrae, as indicated in Table 1. Index level instrumentation was performed in 108 subjects (32.5%), and crosslinks were used in 102 subjects (30.7%).

**Table 1:** Descriptive characteristics of the sample

Variable		Frequency (%)
Sex	Male	204 (61.4)
	Female	128(38.6)
Failure of treatment	Yes	44(13.3)
	No	288(86.7)
Cause of Injury	Road Traffic crashes	181(54.5)
	Fall	106 (31.9)
	Sport	12 (3.6)
	Assault/violence related	29 (8.7)
	Other	4 (1.2)
Level of Vertebra	T10	15 (4.5)
	T11	27 (8.1)
	T12	127 (38.3)
	L1	112 (33.7)
	L2	51 (15.4)
Smoking	Yes	58 (17.5)
	No	274 (82.5)
Diabetes	Yes	54 (16.3)
	No	278(83.7)
Use of crosslinks	Yes	102 (30.7)
	No	230 (69.3)
Index level instrumentation	Yes	108 (32.5)
	No	224(67.5)
Posterolateral fusion	Yes	158 (47.6)
	No	174 (52.4)

Treatment failure occurred in 44 cases (13.3%), primarily due to instrument failure and progressive kyphosis during the follow-up period. All 44 patients underwent long segment posterior spinal fusion during reoperation, and none required a combined anterior-posterior approach (See Table 2).

**Table 2:** Mean and standard deviation of quantitative variables

Variable	Mean	Standard Deviation
Age	47.49	9.75
Follow Up	20.57	5.31
Body Mass Index	23.67	2.09
VBCR (%)	65.33	5.31
PAHC (%)	69.76	5.22
Cobb(°)	13.23	4.07
Canal compromise (%)	21.77	4.57
LSC	5.69	0.81
IPD(%)	18.31	6.42
VAS	5.39	0.68

VBCR: Vertebral body compression rate; PAHC: percentage of anterior height compression; IPD: Interpedicular Distance; VAS: Visual Analogue Scale; LSC: Load sharing classification

Factors associated with treatment failure in the univariate analysis showed that the lack of index level instrumentation, higher BMI, greater Cobb angle and IPD on admission, and a higher LSC score were linked to an increased risk of treatment failure (*p* < 0.05) [Tables 3 and 4]. However, there was no association between treatment failure and age, gender, smoking, VBCR, PAHC, canal compromise, VAS, or the use of crosslinks and posterolateral fusion [Tables 3 and 4].

**Table 3:** Relationship between qualitative variables and failure of treatment

Variable		Failure of treatment		Statistical analysis
		Yes N (%)	No N (%)	
Sex	Male	29 (14.2)	175 (85.8)	*P* = 0.621
	Female	15 (11.7)	113 (88.3)	
Cause of Injury	Road Traffic	28 (15.5)	153 (84.5)	N/A
	Fall	15 (14.2)	91 (85.8)	
	Sport	2 (16.6)	10(83.3)	
	Assault	1 (3.4)	28 (96.6)	
	Other	0(0.00)	4 (100.0)	
Level of Vertebra	T10	3(20.0)	12(80.0)	N/A
	T11	6 (22.2)	21(77.8)	
	T12	19(15.0)	108(85.0)	
	L1	10 (8.9)	102 (91.1)	
	L2	6(11.8)	45 (88.2)	
Smoking	Yes	14(25.9)	40(74.1)	*P* = 0.231
	No	35 (12.8)	239 (87.2)	
Diabetes	Yes	14(25.9)	40 (74.1)	*P* = 0.182
	No	30 (10.8)	248 (89.2)	
Index level instrumentation	Yes	7(6.5)	101 (93.5)	*P* = 0.002
	No	37 (16.5)	187 (83.5)	
Posterolateral fusion	Yes	25(15.8)	133 (84.2)	*P* = 0.163
	No	19 (10.9)	155 (89.1)	
Use of crosslinks	Yes	14 (13.7)	88 (86.3)	*P* = 0.474
	No	30 (13.0)	200 (87.0)	

**Table 4:** Relationship between need for surgery and failure of treatment

Variable	Failure of treatment			Statistical test
	Yes	No		
Age (year)	48.21 (9.91)		47.23 (9.42)	*P* = 0.255
Follow Up	20.37 (5.21)		21.72 (5.42)	*P* = 0.323
Body Mass Index	26.79 (2.2)		23.6 (1.8)	*P = 0.024
VBCR (%)	64.58 (2.39)		66.99 (2.48)	*P* = 0.521
PAHC (%)	68.02 (3.58)		73.16 (4.17)	*P* = 0.235
Cobb(°)	17.11 (3.19)		11.11 (3.09)	*P = 0.013
Canal compromise (%)	24.12 (3.31)		21.22 (3.8)	*P* = 0.221
IPD	27.41 (4.35)		18.88 (3.57)	*p < 0.001
LSC	7.38 (0.79)		4.97 (0.77)	*P < 0.001
VAS	6.11 (0.87)		5.89 (0.79)	*P* = 0.442

VBCR: Vertebral body compression rate; PAHC: percentage of anterior height compression; IPD: Interpedicular Distance; VAS: Visual Analogue Scale; LSC: Load sharing classification

In the multivariate analysis using binary logistic regression, LSC (odds ratio [OR], 2.73; 95% confidence interval [95% CI], 1.31–3.86; *P* = 0.003), the lack of index level instrumentation (OR, 2.33; 95% CI 1.71–3.23; *P* = 0.011), and IPD (OR, 1.84; 95% CI 1.49–2.76; *P* = 0.027) were found to be related to treatment failure [Table 5].

**Table 5:** Binary logistic regression analysis

variables	Odds ratio	95% CI	P value
Index level instrumentation	2.33	1.71–3.23	*P = 0.011
Load sharing classification	2.73	1.31–3.86	*P* = 0.003
Body Mass Index	1.32	0.96–1.64	*P* = 0.327
Cobb (°)	1.151	0.88– 1.72	*P* = 0.428
IPD	1.84	1.49–2.76	*P = 0.027

In this study, each machine learning model utilized feature selection to assess the independent significance of each risk factor. The RF model identified LSC and the lack of index level instrumentation as the most significant variables. The features selected by RF, based on the mean Gini index in descending order, were LSC, the lack of index level instrumentation, IPD, Cobb angle, and BMI.

The study also evaluated the accuracy of LR, RF, SVM, and k-NN models in predicting treatment failure. RF demonstrated the highest accuracy (0.948), followed by SVM (0.933), k-NN (0.927), and LR (0.917) respectively. Additionally, the RF model outperformed other models based on sensitivity and specificity (sensitivity = 0.863, specificity = 0.959). LR, SVM, and k-NN predicted treatment failure with NPVs of 0.857, 0.763, and 0.722 respectively. LR had a PPV of 0.773, followed closely by RF (PPV = 0.748). The AUC values for RF, LR, SVM, and k-NN were 0.911, 0.823, 0.844, and 0.877 respectively [Table 6].

**Table 6:** Evaluation criteria for comparison performance of machine learning models (LR, RF, SVM and k-NN)

Evaluation criteria	Model			
variables	RF	LR	SVM	K-NN
Accuracy	0.948	0.917	0.933	0.927
Sensitivity	0.863	0.697	0.724	0.811
Specificity	0.959	0.945	0.943	0.950
Positive predictive value	0.748	0.773	0.691	0.698
Negative predictive value	0.901	0.857	0.763	0.722
AUC	0.911	0.823	0.844	0.877

RF:Random forest; LR:Logistic regression; SVM:Support vector machine; k-NN: k- nearest neighbor; AUC: area under the curve of mean receiver operating characteristics

## Discussion

Our findings indicate that a higher LSC score, the absence of index level instrumentation, and a greater IPD were linked to treatment failure in patients undergoing SSPSF for traumatic thoracolumbar burst fractures. The primary objective of posterior spinal fixation for these fractures is to restore spinal stability and prevent neurological dysfunction [2, 7]. Previous studies have demonstrated favorable outcomes for SSPSF in carefully selected cases, with significant improvements in kyphosis angle correction and anterior vertebral height [7, 14].

Fracture reduction can be achieved through techniques such as postural reduction, pre-contouring of rods, and cantilever correction. The use of index level instrumentation has been shown to enhance the effectiveness of SSPSF by maintaining sagittal alignment and minimizing instrument failure. Research has indicated that index level instrumentation can substantially increase axial and flexion stiffness, protect the anterior column during flexion-extension loading, and reduce rates of kyphosis correction failure [8, 26].

Additionally, studies have reported that index level instrumentation can protect against correction loss and implant failure, leading to improved kyphosis and vertebral height. However, it is important to note that the presence of a pedicular wall fracture is a contraindication to index level instrumentation, although unilateral pedicle screw insertion on the opposite side of the fractured pedicle has been reported to yield comparable outcomes [27, 28].

Our binary logistic regression analysis revealed that a greater IPD and higher LSC score are associated with an increased risk of treatment failure in patients undergoing SSPSF. The Load Sharing Classification system, developed to quantify vertebral comminution, has been suggested as a prognostic tool for instrumentation failure in SSPSF cases. While some studies have found an association between LSC score and implant failure, others have reported conflicting results [29, 30].

In previous studies, IPD has been identified as an important factor in assessing the severity of thoracolumbar fractures. Greater widening of the interpedicular distance has been linked to a higher likelihood of bone fragment retropulsion into the spinal canal and an increased risk of neurological deficits. IPD has been proposed as a useful parameter for evaluating canal compromise, laminar fractures, and the severity of neurological deficits [31, 32].

The objective of this study was to employ machine learning models to predict factors associated with treatment failure in thoracolumbar burst fractures treated with SSPSF. The results presented in Table 6 indicate that all machine learning models performed well, with Random Forest (RF) demonstrating superior performance across all criteria in predicting treatment failure with the least amount of error. When comparing the classification ability of the evaluated models, RF outperformed the others.

RF is an ensemble learning method that combines multiple decision trees to make predictions. Several characteristics contribute to its superior performance. Firstly, the ensemble approach helps mitigate overfitting and enhances the model’s generalization ability by combining predictions from different subsets of the data. Secondly, RF provides a measure of variable importance, identifying the relative contribution of each input variable in making predictions. This feature aids in identifying influential factors associated with treatment failure. Additionally, RF is capable of capturing complex nonlinear relationships, handling outliers and missing data, and does not assume a specific data distribution, making it suitable for analyzing complex datasets without strict assumptions [33, 34].

The study found that all models demonstrated acceptable performance in terms of the area under the curve (AUC), yielding reliable predictions without sacrificing sensitivity and specificity. However, it was noted that the performance of the predicting models is dependent on the training dataset, and partiality in training can introduce bias. The study used 80% of the data for training and 20% for testing, but acknowledged that a larger dataset would help reduce bias. Missing data was identified as an important limitation, but in this study, there was no missing data due to meticulous physical exams and clinical evaluations.

### Limitations

Limitations of the study should be considered when interpreting the findings and their clinical implications. The retrospective design and reliance on existing medical records may lead to incomplete or missing data, potentially limiting the ability to account for all relevant variables and confounders. Additionally, the study was conducted at a single center, potentially limiting the generalizability of the findings. Although the study included 332 subjects, a larger sample size would enhance statistical power and generalizability.

While the machine learning models demonstrated satisfactory predictive performance, their interpretability may be limited. Understanding the specific factors driving the predictions of these models can be challenging, potentially affecting their clinical utility and decision-making process. Prospective studies with standardized data collection protocols would provide more robust and comprehensive results.

## Conclusions

In conclusion, this study showcased the effectiveness of machine learning models in predicting treatment failure in thoracolumbar burst fractures treated with SSPSF. The results highlight the potential of these models to forecast treatment failure in this specific patient group, providing valuable prognostic insights for early intervention and potential cost reductions.

## Data Availability

No datasets were generated or analysed during the current study.
